# A parabolic correlation between stress hyperglycemia ratio and 28-day mortality in US participants with diabetes and heart failure

**DOI:** 10.1097/MD.0000000000050235

**Published:** 2026-08-21

**Authors:** Qiaowen Huang, Yonggang Peng, Lifang Xiao, Shihan Zhang, Zhiwei Lu, Huan Lin

**Affiliations:** a Department of Anesthesiology, Zhangzhou Affiliated Hospital of Fujian Medical University, Zhangzhou, Fujian Province, China; b Department of Anesthesiology, University of Florida College of Medicine, Gainesville, FL; c Department of Anesthesiology, Zhangzhou Traditional Chinese Medical Hospital, Zhangzhou, Fujian Province, China.

**Keywords:** 28-day all-cause mortality, Medical Information Mart for Intensive Care IV (MIMIC-IV), stress hyperglycemia ratio, U-shaped

## Abstract

Current research lacks sufficient insight into the correlation between the stress hyperglycemia ratio (SHR) and 28-day mortality in critically ill patients with heart failure and diabetes. Our study aims to investigate this correlation. A total of 1697 critically ill patients with heart failure and diabetes were enrolled from the Medical Information Mart for Intensive Care IV 3.1 database. Logistic regression, Cox proportional hazards modeling, and smooth curve fitting analyses were used to meet the research objectives. The total rate of 28-day mortality was 12.1%. Multiple regression analysis showed that the hazard ratios for 28-day mortality in the lowest (Q1), middle (Q3), and highest (Q4) SHR quartiles were 1.85 (95% confidence interval [CI] = 1.17–2.92), 1.38 (95% CI = 0.84–2.20), and 2.52 (95% CI = 1.61–3.93), respectively. Moreover, a U-shaped relationship was found between SHR and mortality risk, with the lowest risk at an SHR of 1.0. Above this level, the hazard ratio increased to 1.707 (95% CI = 1.143–2.549, *P* = .009). Subgroup analysis revealed no other significant findings (*P* > .05). In patients with heart failure and diabetes, SHR exhibits a parabolic association with 28-day all-cause mortality. Notably, an SHR value exceeding 1.0 is associated with a significantly increased risk of 28-day mortality.

## 1. Introduction

Cardiovascular diseases stand as a primary cause of mortality worldwide and are among the principal contributors to disability and death.^[1]^ Heart failure is a critical pathological process in the progression of cardiovascular diseases. Within the United States, the incidence of heart failure falls within the range of 1.9% to 2.6% for the adult population, with a marked increase to 8.5 percent observed in those aged between 65 and 70 years.^[2]^ Diabetes mellitus is closely associated with heart failure. Patients with diabetes mellitus have a 2- to 4-times higher risk of developing heart failure compared with the general population. Moreover, once heart failure is present, the prognosis worsens significantly, with markedly increased mortality and hospitalization rates.^[3,4]^ Diabetes mellitus can lead to structural and functional cardiac abnormalities through multiple mechanisms, including hyperglycemia, insulin resistance, lipotoxicity, oxidative stress, inflammatory response, myocardial fibrosis, and microvascular damage. These alterations can independently trigger so-called “diabetes mellitus cardiomyopathy,” even in the absence of underlying cardiac conditions such as coronary artery disease or hypertension.^[5–7]^

Stress hyperglycemia, characterized by a transient elevation in blood glucose levels due to physiological or psychological stress,^[8,9]^ is a common phenomenon in intensive care unit (ICU) patients. Stress hyperglycemia is closely associated with systemic inflammation, oxidative stress, and endothelial dysfunction, collectively contributing to a poor clinical outcome.^[10–12]^ Research has confirmed an association between hyperglycemia in critically ill patients and increased all-cause mortality, as well as a greater incidence of significant cardiovascular events such as myocardial infarction, heart failure, and stroke.^[13–16]^ The stress hyperglycemia ratio (SHR) reliably predicts poor outcomes in critically ill patients, especially when assessed alongside initial glucose and hemoglobin A1c (HbA1c) levels. This is achieved by evaluating the correlation between stress hyperglycemia severity and disease intensity.^[17–20]^ Previous studies have confirmed that higher SHR values correlate with an increased risk of adverse cardiovascular events such as heart failure and myocardial infarction.^[14]^ Individuals with concomitant heart failure and diabetes are prone to more pronounced blood glucose fluctuations and significant vulnerability to stress-induced hyperglycemia. Nevertheless, prognostic evidence for these patients (particularly those in ICUs) remains limited, and the predictive factors for mortality have yet to be clearly elucidated.

In this retrospective cohort analysis, we included 1687 critically ill patients with congestive heart failure and diabetes, drawing data from the Medical Information Mart for Intensive Care IV (MIMIC-IV) database. The primary objective was to assess the relationship between SHR and the likelihood of 28-day all-cause mortality in these patients upon ICU admission, as well as to elucidate its clinical significance. We also aimed to investigate any potential nonlinear associations between SHR and 28-day all-cause mortality, determining the critical threshold for the SHR’s substantial effect on mortality. These data should aid in the timely recognition of at-risk patients and guide interventions to improve their survival rates.

## 2. Methods

### 2.1. Source of data

This study was approved by the Ethics Committee of Zhangzhou Affiliated Hospital of Fujian Medical University. In this retrospective analysis, we leveraged the recently upgraded MIMIC-IV database (version 3.1) from the Beth Israel Deaconess Medical Center, located in Boston, Massachusetts. This publicly available database encompasses clinical data from patients spanning the years 2008 to 2022.

The dataset includes a wide array of patient information such as demographics, lab results, medication records, vital signs, surgical procedures, diagnoses, treatment plans, and post-discharge survival data. Access to the MIMIC-IV database was granted to author Huan Lin after completing the National Institutes of Health training in human subjects’ research protection and passing the Collaborative Institutional Training Initiative exam (Record ID: 39099161). Given the absence of protected health information and the meticulous anonymization of patient data, informed consent was not required.

### 2.2. Study design and population

**Figure 1.**
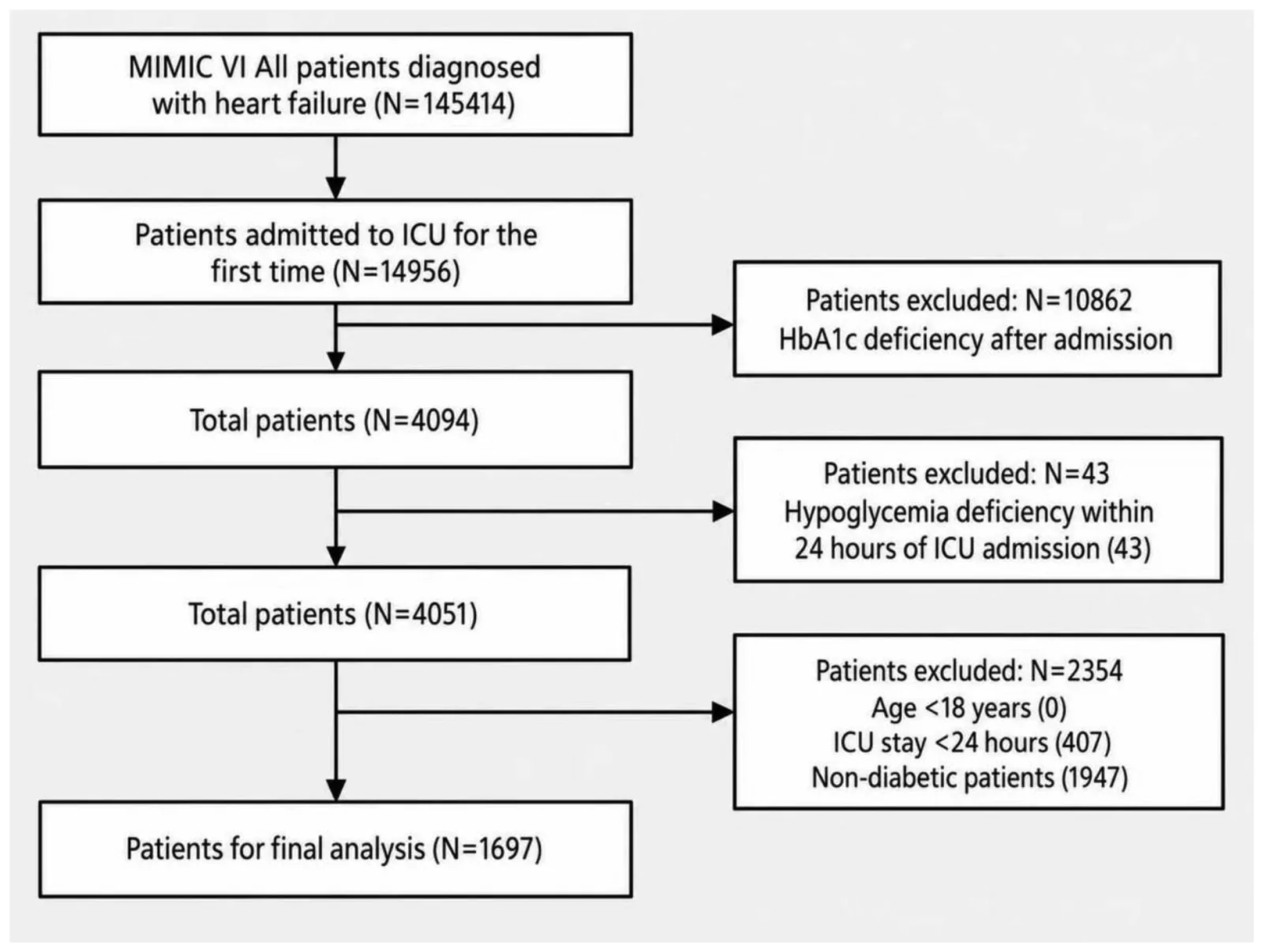
Flowchart of the study cohort. HbA1c = hemoglobin A1c, ICU = intensive care unit, MIMIC-IV = Medical Information Mart for Intensive Care IV.

The study included 14,956 patients initially diagnosed with heart failure who were admitted to an ICU for the first time, identified using International Classification of Diseases (ICD)-9 and ICD-10 codes. The specific codes used for patient selection were as follows: ICD-9: 39891, 40201, 40211, 40291, 40401, 40403, 40411, 40413, 40491, 40493, 4280, 4281, 42820 to 42823, 42830 to 42833, 42840 to 42843, and 4289; and ICD-10: I0981, I110, I130, I132, I502, I5020 to I5023, I503, I5030 to I5033, I504, I5040 to I5043, I508, I5081, I50810 to I50814, I5082 to I5084, I5089, I509, I9713, I97130, and I97131. Patients were excluded if they did not meet the following criteria: absence of HbA1c levels at admission, absence of blood glucose data within the first 24 hours of ICU stay, age <18 years, ICU stay shorter than 24 hours, or no diagnosis of diabetes. After applying these exclusion criteria, 1697 patients were eligible for the study and were recruited and divided into 4 groups according to the quartiles of SHR, as depicted in Figure 1.

### 2.3. Data extraction

Data extraction was facilitated by Navicat Premium software version 16.1.15 (PremiumSoft CyberTech Ltd., Hong Kong, China) in collaboration with structured query language queries. The variables analyzed in this study spanned several key categories, including:

- Demographics: age, gender, weight, Charlson Comorbidity Index, and Sequential Organ Failure Assessment (SOFA) score.
- Medical history: myocardial infarction, peripheral vascular disease, cerebrovascular disease, renal disease, and hypertension.
- Vital signs: heart rate, mean blood pressure, body temperature, and pulse blood oxygen saturation.
- Laboratory indicators: admission glucose (first glucose), HbA1c, hematocrit, hemoglobin, platelets, white blood cell count, anion gap, bicarbonate, blood urea nitrogen, creatinine, sodium, and potassium.
- Length of stay (LOS) and outcomes: LOS in ICU, LOS in hospital, ICU death, hospital death, 28-day all-cause mortality, ventilation, use of vasoactive drugs, and sepsis.

Blood biochemical variables were initially assessed upon patients’ admission to the ICU prior to the initiation of any therapeutic measures. In our analysis, any variables with missing data constituting more than 20% of the dataset were excluded. For those with missing values below this threshold, a multivariate single imputation method was employed for data completion. This approach utilized an iterative imputer, with a Bayesian Ridge model serving as the estimator in each step of the round-robin imputation process.

### 2.4. Calculation of SHR

SHR was calculated with the following equation: SHR = (initial blood glucose level)/([28.7 × HbA1c percentage] − 46.7),^[17]^ where admission glucose and HbA1c values were extracted directly from the MIMIC-IV database.

### 2.5. Outcomes

The primary outcome of the current research investigation was 28-day all-cause mortality.

### 2.6. Statistical analyses

Variables with a normal distribution were characterized by their mean and standard deviation, while those without were represented by the median and interquartile range. Categorical data were expressed as frequencies and percentages. To analyze variations among different SHR groups, we used various statistical methods: chi-square or Fisher’s exact tests for categorical variables, one-way analysis of variance for normally distributed data, and the Kruskal–Wallis *H* test for data lacking normality.

We employed multivariate Cox proportional hazards regression models to estimate the hazard ratios (HRs) and their respective 95% confidence intervals (CIs) for 28-day all-cause mortality. For participants with incomplete follow-up data, right-censoring was applied at the time of their last follow-up. The survival analysis for 28-day all-cause mortality was conducted using Kaplan–Meier survival curves, which were stratified based on categorical SHR, and differences between groups were assessed using the log-rank test. The confounding variables included in the models were selected based on their clinical significance, alignment with existing literature, and their statistical significance in the univariate analysis. Specifically, Model I was adjusted for age and gender; Model II incorporated further adjustments for weight, history of myocardial infarction, hypertension, cerebrovascular disease, and SOFA score; Model III expanded the adjustment to include arterial blood pressure, total ventilation index, use of vasoactive drugs, serum creatinine levels, white blood cell count, and hemoglobin levels.

Each SHR was stratified into 4 quartiles, and trend analysis was performed using *P*-values to validate its linear relationship as a continuous variable and to explore potential nonlinear associations with 28-day all-cause mortality. Restricted cubic spline models were employed to generate smoothed curves, identifying potential nonlinear relationships between SHR and 28-day all-cause mortality. SHR was modeled as a continuous variable with 4 knots placed at the 5th, 35th, 65th, and 95th percentiles, following Harrell’s recommendations. Nonlinearity was assessed using a likelihood ratio test, comparing a linear-only model with a model incorporating both linear and cubic spline components. Where nonlinear associations were identified, a piecewise regression model was applied to delineate the threshold effects as suggested by the smoothing plot. Subgroup analyses were conducted to explore interactions. Continuous variables were categorized using clinical cutoffs or quartiles, followed by an interaction test. Missing data were addressed through simple imputation. Sensitivity analyses were conducted to evaluate the stability of our findings and to determine the influence of various association inference models on our conclusions. We also performed stratified analyses based on gender (male and female), age (<71.9 years or 71.9 years and above), and history of myocardial infarction. The effect sizes and corresponding *P*-values from all models were meticulously calculated, documented, and compared.

Statistical analyses were conducted using R version 4.2.2 (R Foundation for Statistical Computing) and the Free Statistics Analysis Platform version 1.9.2 (Beijing Fengrui Kelin Medical Technology Co., Ltd.). We defined statistical significance as a two-tailed *P*-value ≤ .05, with α set at 0.05.

## 3. Results

### 3.1. Patient demographics and baseline characteristics across SHR quartiles

Figure 1 illustrates the participant selection flowchart, culminating in the inclusion of 1697 patients with comprehensive respiratory data. This cohort (aged 68.9–70.5 years) was stratified into 4 distinct groups based on SHR quartiles: Q1 (<0.894), Q2 (0.894–1.105), Q3 (1.105–1.368), and Q4 (≥1.368). Demographic and initial characteristics of these cohorts are detailed in Table 1. Patients in the highest SHR quartile (Q4) exhibited significantly higher heart rates, elevated blood glucose levels, a greater prevalence of myocardial infarction history, and a higher incidence of sepsis. They also had increased use of vasopressors and longer ICU stays, along with higher rates of ICU mortality, in-hospital mortality, and 28-day all-cause mortality compared with other quartiles. Additionally, they displayed higher white blood cell counts, anion gaps, blood urea nitrogen and creatinine levels, and lower body weight, bicarbonate, sodium, and glycated hemoglobin levels (all *P* < .05).

**Table 1:** Patient demographics and baseline characteristics across different SHR groups.

Characteristic	Total (n = 1697)	SHR, Q1 (<0.894)	SHR, Q2 (0.894–1.105)	SHR, Q3 (1.105–1.368)	SHR, Q4 (≥1.368)	*P*-value
		n = 514	n = 356	n = 375	n = 452	
Demographics						
Age, yr	70.2 ± 11.6	68.9 ± 11.6	70.3 ± 11.9	71.6 ± 11.2	70.5 ± 11.5	.009
Weight, kg	90.2 ± 25.5	92.0 ± 26.5	92.2 ± 25.9	88.4 ± 24.9	88.1 ± 24.3	.023
Gender, n (%)						.063
Female	648 (38.2)	183 (35.6)	123 (34.6)	159 (42.4)	183 (40.5)	
Male	1049 (61.8)	331 (64.4)	233 (65.4)	216 (57.6)	269 (59.5)	
Vital signs						
Heart rate, beats/min	84.0 ± 14.2	83.6 ± 13.8	82.1 ± 12.9	84.0 ± 14.0	85.8 ± 15.5	.002
MBP, mm Hg	77.5 ± 10.6	77.6 ± 11.0	77.9 ± 11.1	76.6 ± 9.2	77.9 ± 10.9	.232
Body temperature, °C	36.8 ± 0.5	36.8 ± 0.5	36.8 ± 0.5	36.9 ± 0.5	36.8 ± 0.5	.119
SpO_2_, %	97.0 ± 1.9	97.0 ± 1.9	97.1 ± 1.8	97.0 ± 1.9	96.8 ± 1.9	.142
Laboratory indicators						
Hematocrit, vol %	30.2 ± 6.9	30.5 ± 7.1	29.7 ± 7.0	30.0 ± 6.6	30.4 ± 6.9	.349
Hemoglobin, g/dL	9.8 ± 2.3	9.9 ± 2.3	9.7 ± 2.3	9.7 ± 2.1	9.9 ± 2.4	.317
Platelets, 10^9^/L	185.0 ± 83.3	189.0 ± 83.1	170.2 ± 72.5	182.3 ± 78.2	194.4 ± 93.6	<.001
WBC count, 10^9^/L	15.0 ± 7.0	14.5 ± 6.6	14.1 ± 6.3	14.8 ± 5.9	16.4 ± 8.3	<.001
Anion gap, mmol/L	16.4 ± 5.0	15.9 ± 4.7	15.4 ± 4.6	16.0 ± 4.5	18.2 ± 5.5	<.001
Bicarbonate, mmol/L	21.5 ± 4.8	22.2 ± 4.9	21.9 ± 4.2	21.7 ± 4.3	20.4 ± 5.2	<.001
BUN, mg/dL	36.1 ± 24.4	33.8 ± 22.7	34.8 ± 24.3	33.7 ± 22.7	41.6 ± 26.7	<.001
Creatinine, mg/dL	2.0 ± 1.8	1.9 ± 1.5	2.0 ± 1.9	1.9 ± 1.8	2.3 ± 2.0	.002
Sodium, mmol/L	136.0 ± 4.6	136.2 ± 4.7	136.6 ± 4.2	136.1 ± 4.1	135.2 ± 5.2	<.001
Potassium, mmol/L	4.8 ± 0.9	4.8 ± 0.8	4.8 ± 0.9	4.8 ± 0.8	4.9 ± 0.9	.053
First HbA1c, %	7.5 ± 1.9	8.3 ± 2.1	7.2 ± 1.5	7.2 ± 1.7	7.1 ± 1.8	<.001
First glucose, mg/dL	193.0 ± 97.3	130.5 ± 44.5	160.1 ± 46.3	197.2 ± 60.1	286.4 ± 121.0	<.001
Past medical history						
Myocardial infarction, n (%)						.003
No	844 (49.7)	280 (54.5)	181 (50.8)	190 (50.7)	193 (42.7)	
Yes	853 (50.3)	234 (45.5)	175 (49.2)	185 (49.3)	259 (57.3)	
Peripheral vascular disease, n (%)						.397
No	1415 (83.4)	438 (85.2)	300 (84.3)	305 (81.3)	372 (82.3)	
Yes	282 (16.6)	76 (14.8)	56 (15.7)	70 (18.7)	80 (17.7)	
Cerebrovascular disease, n (%)						.688
No	1269 (74.8)	381 (74.1)	261 (73.3)	280 (74.7)	347 (76.8)	
Yes	428 (25.2)	133 (25.9)	95 (26.7)	95 (25.3)	105 (23.2)	
Renal disease, n (%)						.057
No	913 (53.8)	286 (55.6)	178 (50)	219 (58.4)	230 (50.9)	
Yes	784 (46.2)	228 (44.4)	178 (50)	156 (41.6)	222 (49.1)	
Hypertension, n (%)						.87
No	1296 (76.4)	393 (76.5)	269 (75.6)	283 (75.5)	351 (77.7)	
Yes	401 (23.6)	121 (23.5)	87 (24.4)	92 (24.5)	101 (22.3)	
Disease severity score						
Charlson Comorbidity Index	8.4 ± 2.4	8.3 ± 2.4	8.5 ± 2.4	8.4 ± 2.4	8.6 ± 2.3	.057
SOFA score	5.5 ± 3.1	5.4 ± 3.1	5.5 ± 3.1	5.4 ± 3.0	5.7 ± 3.3	.417
Sepsis, n (%)						<.001
No	783 (46.1)	258 (50.2)	172 (48.3)	188 (50.1)	165 (36.5)	
Yes	914 (53.9)	256 (49.8)	184 (51.7)	187 (49.9)	287 (63.5)	
Outcomes						
Length of stay (LOS)						
LOS in ICU (IQR)	3.1 (1.9, 5.7)	3.2 (1.9, 5.9)	3.0 (1.9, 5.2)	3.1 (1.9, 5.3)	3.3 (2.0, 6.2)	.169
LOS in hospital (IQR)	11.8 (7.7, 17.9)	11.9 (7.7, 17.8)	12.1 (8.0, 18.0)	11.0 (7.9, 18.0)	11.7 (7.0, 17.8)	.614
ICU death, n (%)						.004
No	1579 (93.0)	479 (93.2)	341 (95.8)	354 (94.4)	405 (89.6)	
Yes	118 (7.0)	35 (6.8)	15 (4.2)	21 (5.6)	47 (10.4)	
Hospital death, n (%)						<.001
No	1514 (89.2)	462 (89.9)	332 (93.3)	340 (90.7)	380 (84.1)	
Yes	183 (10.8)	52 (10.1)	24 (6.7)	35 (9.3)	72 (15.9)	
28-day all-cause mortality, n (%)						<.001
No	1491 (87.9)	451 (87.7)	330 (92.7)	338 (90.1)	372 (82.3)	
Yes	206 (12.1)	63 (12.3)	26 (7.3)	37 (9.9)	80 (17.7)	
Ventilation, n (%)						.801
No	720 (42.4)	215 (41.8)	153 (43)	153 (40.8)	199 (44)	
Yes	977 (57.6)	299 (58.2)	203 (57)	222 (59.2)	253 (56)	
Use of vasoactive drugs, n (%)						.038
No	907 (53.4)	253 (49.2)	183 (51.4)	215 (57.3)	256 (56.6)	
Yes	790 (46.6)	261 (50.8)	173 (48.6)	160 (42.7)	196 (43.4)	

Data are expressed as mean ± standard deviation, median (interquartile range), or n (%).

BUN = blood urea nitrogen, HbA1c = hemoglobin A1c, ICU = intensive care unit, IQR = interquartile range, MBP = mean blood pressure, SHR = stress hyperglycemia ratio, SOFA = Sequential Organ Failure Assessment, SpO_2_ = peripheral oxygen saturation, WBC = white blood cell.

Conversely, there were no notable disparities detected among the quartiles in weight, gender, mean blood pressure, body temperature, peripheral oxygen saturation, ICU LOS, hospital LOS, peripheral vascular disease, cerebrovascular disease, hypertension, Charlson Comorbidity Index, SOFA score, ventilation, and renal disease (all *P* > .05).

### 3.2. Outcomes

The overall 28-day all-cause mortality rate among the study participants was 12.1%. Specifically, the mortality rates for groups Q1, Q2, Q3, and Q4 were 12.3%, 7.3%, 9.9%, and 17.7%, respectively.

### 3.3. SHR and 28-day all-cause mortality

**Figure 2.**
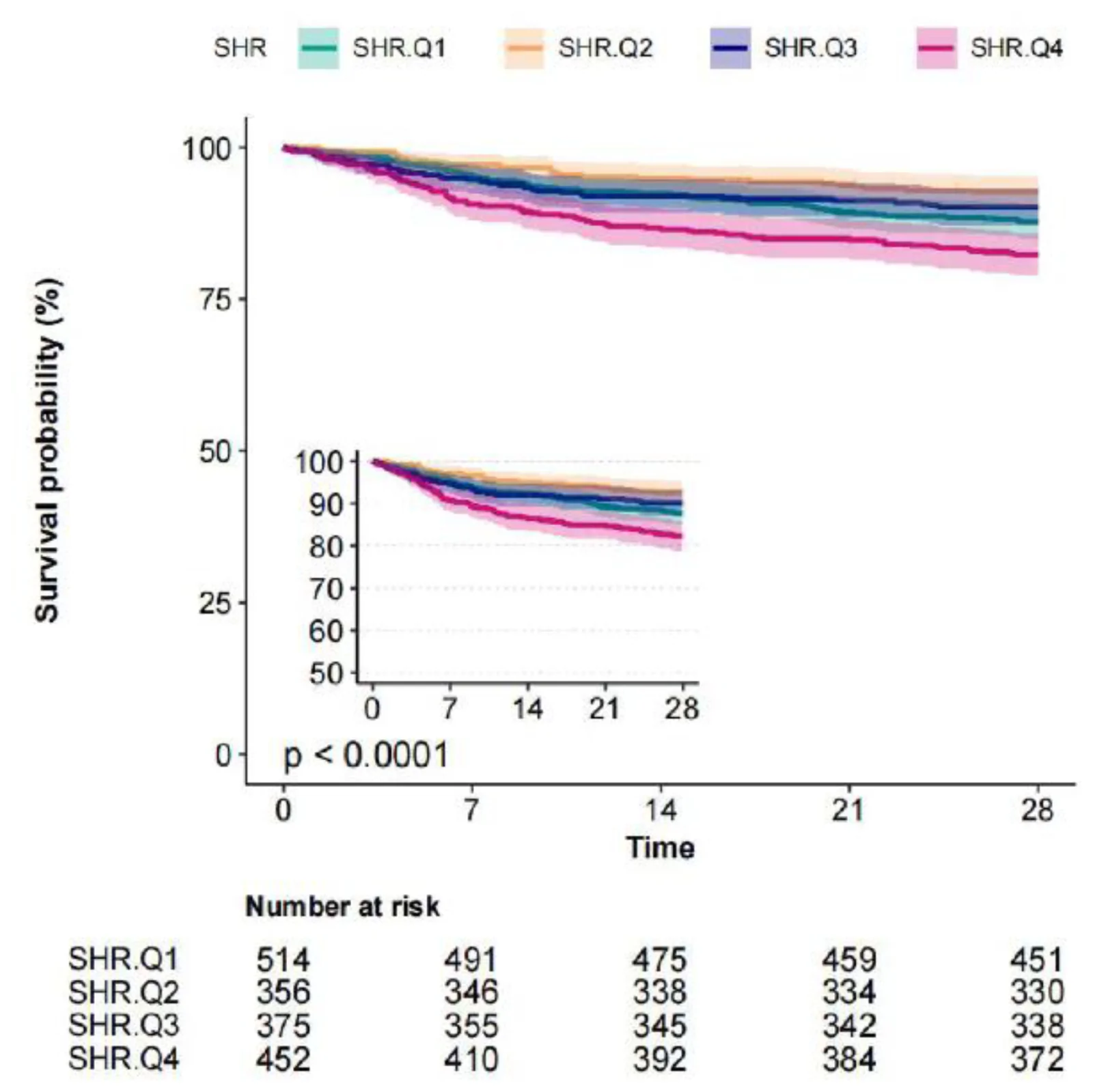
Kaplan–Meier curves of 28-day all-cause mortality stratified by SHR quartiles. The Kaplan–Meier curve compares the 28-day survival probability of the entire cohort and groups stratified by SHR quartiles (Q1: green; Q2: orange; Q3: purple; Q4: pink). The shaded areas around the survival curves represent the 95% confidence intervals. A magnified view of the first 28 days is provided in the inset. The number of subjects at risk at each time point for every group is detailed in the table below the graph. The log-rank test *P*-value (*P* < .0001) indicates a statistically significant difference in survival distributions among the groups. SHR = stress hyperglycemia ratio.

Univariate analysis revealed a significant correlation between SHR and 28-day all-cause mortality, with an HR of 1.45 (95% CI, 1.19–1.76; *P* < .001; Table 2). This association was further supported by Kaplan–Meier survival curves, which showed a statistically significant difference in survival distributions (log-rank test *P* < .05; Fig. 2). In multivariate Cox proportional hazards modeling, SHR, treated as a categorical variable, exhibited a positive correlation with the risk of 28-day all-cause mortality, particularly when comparing Q4 with Q2, with an HR of 2.58 (95% CI, 1.66–4.02; *P* < .001). After adjusting for potential confounders, this correlation remained robust, with an HR of 2.52 (95% CI, 1.61–3.93; *P* < .001), as detailed in Table 3, Model III.

**Table 2:** Univariate analysis for 28-day all-cause mortality.

Variables	Hazard ratio (95% CI)	*P*-value (Wald’s test)
Age, yr	1.04 (1.03–1.06)	<.001
Gender: male vs female	0.89 (0.67–1.18)	.412
Weight, kg	0.9932 (0.9874–0.999)	.022
MBP, mm Hg	0.99 (0.97–1)	.039
Heart rate (beats/min)	1.0094 (1–1.019)	.051
Myocardial infarction: yes vs no	1.09 (0.83–1.43)	.546
Hypertension: yes vs no	0.55 (0.38–0.81)	.002
Peripheral vascular disease: yes vs no	1.15 (0.81–1.64)	.425
Cerebrovascular disease: yes vs no	2.2 (1.67–2.9)	<.001
Use of vasoactive drugs: yes vs no	1.35 (1.02–1.77)	.034
Ventilation: yes vs no	1.44 (1.08–1.92)	.013
Creatinine (mg/dL)	1.11 (1.05–1.18)	<.001
WBC (10^9^/L)	1.03 (1.01–1.04)	.003
Hemoglobin (g/dL)	0.97 (0.91–1.03)	.312
SOFA score	1.13 (1.08–1.18)	<.001
SHR	1.45 (1.19–1.76)	<.001

In univariate analysis, age, weight, MBP, heart rate, cerebrovascular disease, use of vasoactive drugs, ventilation, creatinine, WBC count, SOFA score, and SHR were significantly associated with 28-day all-cause mortality, whereas gender, myocardial infarction, hypertension, peripheral vascular disease, and hemoglobin were not significantly associated with 28-day all-cause mortality.

CI = confidence interval, MBP = mean blood pressure, SHR = stress hyperglycemia ratio, SOFA = Sequential Organ Failure Assessment, WBC = white blood cell.

**Table 3:** Multivariate Cox regression for SHR on 28-day all-cause mortality.

Variables	Non-adjusted Model		Model I		Model II		Model III	
	HR (95% CI)	*P*-value	HR (95% CI)	*P*-value	HR (95% CI)	*P*-value	HR (95% CI)	*P*-value
SHR quartile								
Q1 (<0.894)	1.72 (1.09–2.71)	.021	1.83 (1.16–2.89)	.01	1.87 (1.18–2.96)	.007	1.85 (1.17–2.92)	.009
Q2 (0.894–1.105)	1 (Reference)		1 (Reference)		1 (Reference)		1 (Reference)	
Q3 (1.105–1.368)	1.38 (0.84–2.29)	.203	1.35 (0.81–2.22)	.246	1.38 (0.84–2.28)	.209	1.38 (0.84–2.29)	.205
Q4 (≥1.368)	2.58 (1.66–4.02)	<.001	2.6 (1.67–4.04)	<.001	2.57 (1.65–4.01)	<.001	2.52 (1.61–3.93)	<.001

Model I: adjusted for gender + age.

Model II: adjusted for Model I + weight + myocardial infarction + hypertension + cerebrovascular disease + SOFA.

Model III: adjusted for Model II + MBP + ventilation + use of vasoactive drugs + creatinine + WBC + hemoglobin.

CI = confidence interval, HR = hazard ratio, MBP = mean blood pressure, SHR = stress hyperglycemia ratio, SOFA = Sequential Organ Failure Assessment, WBC = white blood cell.

### 3.4. U-shaped association of SHR with 28-day all-cause mortality

**Figure 3.**
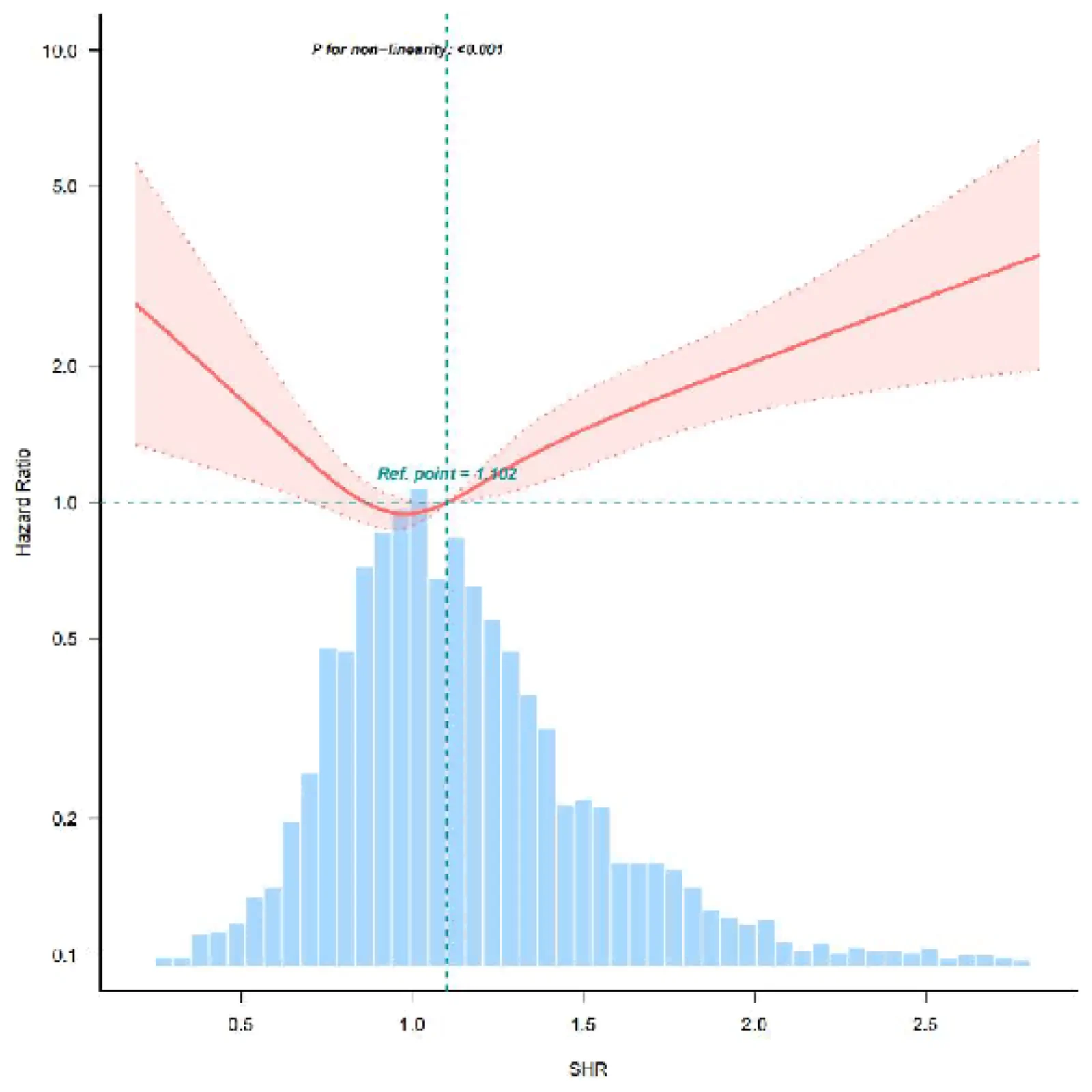
Illustrates the association between SHR levels and 28-day all-cause mortality. The data were adjusted for gender, age, weight, and other variables (Model III). The reference standard for SHR was set at the median value. The shaded area represents the 95% confidence interval. Only 99% of the data are shown. SHR = stress hyperglycemia ratio.

This study employed a smoothing function analysis to explore the dose-response relationship between SHR levels and the risk of 28-day all-cause mortality. After controlling for potential confounders, a significant nonlinear association was observed between SHR levels and mortality risk (*P* for nonlinearity = .002; Fig. 3). The risk of 28-day all-cause mortality decreased with increasing SHR levels, reaching a nadir at an SHR of 1.0, with an HR of 0.151 (95% CI, 0.046–0.498; *P* = .0019). Notably, beyond an SHR of 1.0, the HR for mortality risk increased to 1.707 (95% CI, 1.143–2.549), indicative of a parabolic association. This pattern suggests that both very low and very high SHR levels are linked to higher risks of 28-day all-cause mortality, as further detailed in Table 4.

**Table 4:** Threshold effect analysis of SHR on the level and risk of 28-day all-cause mortality.

Threshold of SHR	HR	95% CI	*P*-value
<1.0	0.151	0.046–0.498	.0019
≥1.0	1.707	1.143–2.549	.009
Likelihood ratio test		–	<.001

Adjusted for sociodemographic (Model III: adjusted for gender + age + weight + myocardial infarction + hypertension + cerebrovascular disease + SOFA + MBP + ventilation + use of vasoactive drugs + creatinine + WBC + hemoglobin). Only 99% of the data are displayed.

CI = confidence interval, HR = hazard ratio, MBP = mean blood pressure, SHR = stress hyperglycemia ratio, SOFA = Sequential Organ Failure Assessment, WBC = white blood cell.

### 3.5. Subgroup analyses

**Figure 4.**
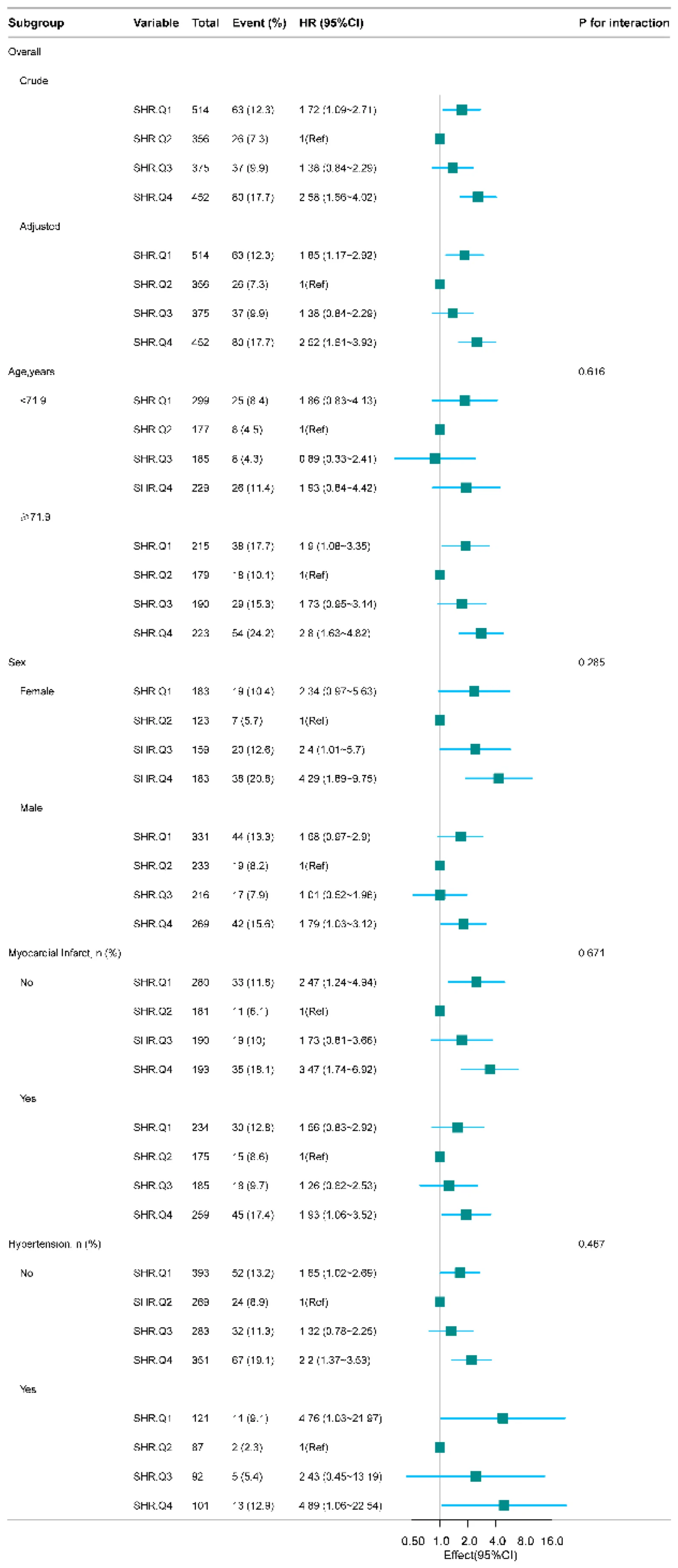
Subgroup analysis and forest plot for age, gender, myocardial infarction, and hypertension based on model III. Hazard ratios (HRs) with 95% confidence intervals (CIs) are presented for both crude and adjusted models, as well as across various prespecified subgroups. The analysis is stratified by SHR quartiles (Q1–Q4), with Q2 serving as the reference group (Ref). The size of the data markers corresponds to the weight of each subgroup in the analysis. The vertical solid line indicates the null effect (HR = 1.0). Horizontal lines represent the 95% CIs. The *P*-values for interaction were calculated to assess the heterogeneity of the treatment effect across subgroups. In each case, the model was not adjusted for the stratification variable. SHR = stress hyperglycemia ratio.

We performed multivariable stratified analyses across various patient subgroups, including those defined by age, gender, history of myocardial infarction, and hypertension. No significant interaction effects were observed, as all interaction *P*-values exceeded .05 (Fig. 4).

## 4. Discussion

In this retrospective cohort analysis utilizing the MIMIC-IV database, a parabolic relationship was identified between SHR and the risk of 28-day all-cause mortality among patients with concomitant heart failure and diabetes, as determined by restricted cubic spline analysis (*P* = .002). The nadir of this risk was observed at an SHR value of 1.0. For SHR values below 1.0, the HR for 28-day all-cause mortality was 0.151 (95% CI, 0.046–0.498; *P* = .0019). Conversely, for SHR values above 1.0, the HR increased to 1.707 (95% CI, 1.143–2.549; *P* = .009). This pattern was consistently replicated in subgroup analyses. The clinical implications of these findings are significant.

Prior research has established that SHR is linked not only to the risks of cerebral edema and infection in patients hospitalized following an acute cerebral infarction^[19,21]^ but also to the incidence of major adverse cardiovascular events.^[22]^ These associations underscore SHR’s potential as a significant clinical prognostic marker.

Additionally, individuals with a history of diabetes, particularly those with concomitant endothelial dysfunction – a condition that negatively affects vascular function – tend to exhibit more pronounced hypoglycemic episodes and a significant vulnerability to ischemic events.^[23]^ Such arrhythmias and ischemic incidents frequently result in the deterioration of cardiac function, ultimately contributing to mortality.

Recent research has indicated that the assessment of SHR and glycemic variability enhances risk stratification in patients with coronary heart disease, facilitates personalized blood glucose management, and subsequently leads to improved patient outcomes.^[24]^ Additionally, a separate study from China proposes a parabolic correlation between SHR and multiple mortality outcomes – including all-cause mortality, cardiovascular mortality, and heart failure readmission – in diabetic patients with acute heart failure.^[9]^ It is important to note that this study’s findings may be subject to ethnic variations and are specific to the context of acute heart failure.

In this investigation, we leveraged data from the MIMIC-IV database to include all critically ill patients with heart failure and diabetes, thereby achieving a more robust sample size. Our univariate and multivariate analyses revealed a significant positive correlation between SHR and 28-day mortality among these patients. Furthermore, by categorizing patients into quartiles based on SHR and accounting for potential confounders – including age, gender, comorbidities, and laboratory parameters – we consistently identified a parabolic association with 28-day mortality. These findings bolster the current body of knowledge, suggesting that both extremely low and high SHR values are linked to an elevated risk of 28-day all-cause mortality. Consequently, SHR may emerge as a potent predictor of 28-day mortality.

The precise mechanisms linking stress hyperglycemia to heart failure and its impact on the long-term prognosis of heart failure remain incompletely elucidated. Research indicates that transient spikes in blood glucose can trigger endothelial dysfunction, oxidative stress, inflammation, and coagulation cascades, which may precipitate atherosclerosis and cardiomyopathy. These pathophysiological alterations can compromise myocardial contractility, enhance fluid retention, exacerbate the symptoms of heart failure, and increase the mortality risk.^[11,25,26]^ Conversely, a very low SHR correlates with adverse long-term outcomes in patients with heart failure, potentially due to an increased frequency of hypoglycemic episodes, which are known to elevate the risk of mortality and cardiovascular events.^[27,28]^ The relationship between SHR and all-cause mortality in heart failure is thought to be significantly influenced by the inflammatory responses to blood glucose fluctuations.^[29]^

Notably, the significant association between SHR and 28-day mortality demonstrated a moderate yet consistent effect across various event subtypes, such as those stratified by age, gender, history of myocardial infarction, and hypertension. While it is plausible that SHR could serve as a predictor for adverse outcomes, this hypothesis awaits confirmation through future research endeavors.

Despite our best efforts, there are still some limitations to this study. To begin with, the assessment of blood glucose levels and HbA1c was limited to the initial time point, and the SHR was determined on a 1-time basis. This method might not adequately represent the fluctuations in blood sugar regulation that occur longitudinally. Future research should incorporate repeated assessments of SHR and glycemic parameters to enhance our understanding of these relationships. Secondly, despite our efforts to account for all pertinent confounders in the multivariate analysis, there remains the possibility of unmeasured or residual confounding factors, such as socioeconomic status, which includes variables like income, education level, and disparities in healthcare access, potentially influencing patient outcomes and therapeutic decisions. The homogeneity of our study population, drawn from a single hospital, may also limit the generalizability of our findings. While we controlled for several significant confounders, the influence of unknown factors cannot be entirely ruled out and may affect our results. Therefore, caution is warranted when interpreting the risk estimates presented in this study, as the biological plausibility of our findings requires further validation, and data imputation could introduce spurious associations. Additional studies are essential to corroborate these preliminary results and to discern any random findings. Nonetheless, the conduct of well-designed, multicenter, controlled trials remains essential for the validation of our results.

## 5. Conclusion

The association between SHR and the risk of 28-day all-cause mortality exhibits a parabolic curve among patients with concomitant heart failure and diabetes. Notably, an SHR value exceeding 1.0 is associated with an elevated risk of 28-day all-cause mortality. Further validation and confirmation of these results are essential to bolster their reliability and ensure their practical application in clinical practice.

## Acknowledgments

We extend our sincere appreciation to the dedicated staff and fellow researchers for their valuable contributions. We are particularly grateful to Bryan Penberthy, MFA, MWC, of the University of Florida College of Medicine Department of Anesthesiology’s Communications & Publishing office for his editorial assistance with this manuscript.

## Author contributions

**Conceptualization:** Qiaowen Huang, Yonggang Peng, Lifang Xiao, Shihan Zhang, Zhiwei Lu, Huan Lin.

**Data curation:** Qiaowen Huang, Yonggang Peng, Lifang Xiao, Shihan Zhang, Zhiwei Lu, Huan Lin.

**Formal analysis:** Qiaowen Huang, Yonggang Peng, Lifang Xiao, Shihan Zhang, Zhiwei Lu, Huan Lin.

**Funding acquisition:** Qiaowen Huang, Yonggang Peng, Lifang Xiao, Shihan Zhang, Zhiwei Lu, Huan Lin.

**Investigation:** Qiaowen Huang, Huan Lin.

**Writing – original draft:** Huan Lin.

**Writing – review & editing:** Huan Lin.
